# Attitudes Towards Suicide Questionnaire (ATTS): translation and adaptation to the Brazilian context

**DOI:** 10.1590/0034-7167-2024-0575

**Published:** 2025-09-05

**Authors:** Bruna Marques Chiarelo, Laysa Fernanda Silva Pedrollo, Micael Doria de Andrade, Ana Carolina Guidorizzi Zanetti, Kelly Graziani Giacchero Vedana

**Affiliations:** I Universidade de São Paulo. Ribeirão Preto, São Paulo, Brazil

**Keywords:** Attitude, Suicide, Translating, Methodological Studies, Suicide Prevention., Actitud, Suicidio, Traducción, Estudio Metodológico, Prevención del Suicidio.

## Abstract

**Objectives:**

to translate and adapt the Attitudes Towards Suicide questionnaire to the Brazilian context.

**Methods:**

methodological, cross-sectional study. The original English version of the instrument was translated into Brazilian Portuguese, subsequently back-translated into the original language and sent to the original authors. It was then assessed by a Committee of Experts composed of nine members and evaluated through a pre-test with 35 participants from the general population aged 18 to 65 years. Data were coded and analyzed using a statistical software.

**Results:**

most participants evaluated the questionnaire as good (88.6%); with important items (94.3%); easy to understand (88.6%) and without need for changes (91.2%).

**Conclusions:**

the instrument allows the analysis of factors that may influence attitudes towards suicide in the national context. This information may support better interventions and strategic actions for suicide prevention.

## RESUMEN

**Objetivos:**

traducir y adaptar el cuestionario de Actitudes Hacia el Suicidio al contexto brasileño.

**Métodos:**

estudio metodológico transversal. La versión original del instrumento, en inglés, se tradujo al portugués brasileño, se retrotradujo al idioma original y se envió a los autores originales. Posteriormente, fue evaluado por un Comité de Expertos compuesto por nueve miembros y se evaluó mediante una prueba previa con 35 participantes de la población general de 18 a 65 años. Los datos se codificaron y analizaron mediante un programa estadístico.

**Resultados:**

la mayoría de los participantes evaluaron el cuestionario como bueno (88,6%); con elementos importantes (94,3%); fácil de entender (88,6%) y sin necesidad de cambios (91,2%).

**Conclusiones:**

el instrumento permite el análisis de los factores que pueden influir en las actitudes hacia el suicidio en el contexto nacional. Esta información puede apoyar mejores intervenciones y acciones estratégicas para la prevención del suicidio.

## INTRODUCTION

Suicide is defined as self-inflicted death with evidence that the person intended to die^(1)^. The phenomenon is complex, multifactorial and a major public health problem^(2)^. It is estimated that 703,000 people die annually by suicide^(3)^. In Brazil, the suicide rate is 6.4 per 100,000 inhabitants and according to global estimates, 14,540 suicides were recorded in 2019^(3)^.

Attitudes related to suicide can interfere with behaviors of asking for and offering help^(4)^. Available evidence has shown that these attitudes can vary according to sociodemographic characteristics such as gender, ethnicity, culture, experiences with suicide of family members and significant others, expressions of suicidal behavior throughout life, age^(5-7)^ and religious beliefs^(8,9)^. However, the association between professional training related to suicide and better attitudes is frequently identified^(4)^.

The assessment of these attitudes can help in the development of policies and strategies aimed at prevention^(10)^, and in the application of consistent interventions within the context and unique conditions experienced by groups with specific needs and resources, enabling more assertive, diverse, inclusive and equitable planning^(6)^. The various factors that interfere in attitudes highlight the challenges in measuring the construct in a thorough and comprehensive manner^(11,12)^.

The *Questionário sobre Atitudes Frente ao Comportamento Suicida* (QACS, Questionnaire on Attitudes Towards Suicidal Behavior) was the only instrument available in the Brazilian context with this focus. However, its use is restricted to health professionals and the instrument has limitations related to the restricted assessment of a few dimensions of attitudes towards suicide, little practicality in obtaining scores (manual conversion of the visual scale into a score) and the uselessness of some items of the scale for calculating factor scores^(13)^.

The Attitudes Towards Suicide (ATTS) questionnaire stands out in the international context as a widely used instrument that considers the multidimensional aspects of suicide-related attitudes. This instrument was developed by Renberg and Jacobsson^(14)^ based on the Suicide Opinion Questionnaire (SOQ) and the Suicide Attitude Questionnaire (SUIATT)^(15-17)^. Its widespread use is explained by the number of items, the ten factors assessed, the dimensions involved, and the satisfactory psychometric characteristics presented by the instrument^(17)^.

The ATTS has been adapted and validated for different countries such as Sweden, Norway, Russia^(6)^, India^(5)^, Malaysia^(18)^, Japan^(19)^, South Korea^(10)^, Poland^(17)^, Iran^(20)^ and has been used as an important instrument to assess suicide-related attitudes in various populations. The lack of evidence of the adaptation of the ATTS to the Brazilian context reinforces the possibility of making it available to assess attitudes toward suicide in the country.

## OBJECTIVES

To translate and adapt the Attitudes Towards Suicide questionnaire to the Brazilian context.

## METHODS

### Translation and back-translation

A methodological study of translation and cross-cultural adaptation. The development of the study was approved by the Research Ethics Committee of the Escola de Enfermagem de Ribeirão Preto at the *Universidade de São Paulo* (EERP/USP). Authorization from the original authors of the instrument was obtained prior to conducting the study.

The 34-item version of the ATTS was adapted and validated. This version presents a Likert-type scale of 1 to 5 points ranging from “Strongly Disagree” to “Strongly Agree”. The greater the agreement the higher the score, except for items 7 and 9, evaluated in reverse order. The instrument assesses 10 factors, namely: Suicide as a right (Factor 1), Incomprehensibility (Factor 2), Noncommunication (Factor 3), Preventability (Factor 4), Tabooing (Factor 5), Normal-common (Factor 6), Suicide as a process (Factor 7), Relation-caused (Factor 8), Preparedness to prevent (Factor 9), and Suicides as solution or resignation (Factor 10). The internal consistency of the items ranges from 0.38 to 0.86, and of the complete instrument is 0.60^(14)^.

The process of cross-cultural adaptation of the study followed the recommendations in the literature^(21,22)^. To obtain the first consensual version in Portuguese, two translations from English into Portuguese were carried out independently by two bilingual translators, familiar with both cultures and whose native language was Brazilian Portuguese. They were instructed on the need to use clear and simple language^(21)^. The two versions produced were compared in a meeting with the translators to create a consensual version in Portuguese. The translators received guidance on suicide-related safe communication to avoid stigmatizing or contraindicated expressions such as “commit suicide”, “complete suicide”, among others^(23)^.

In order to assess inconsistencies, conceptual equivalence and quality of the translation, the consensual version in Portuguese was back-translated (into English) independently by two native, bilingual translators whose native language was English^(21)^. The two versions of the back-translation were compared in a meeting with the translators to create a final consensual version of the back-translation into English. The version created was sent to the original authors of the study for assessment in relation to possible inconsistencies, considering the original proposal of the ATTS and its purpose. Three emails were sent to the original authors of the ATTS asking them to indicate any changes needed and they were informed that if a response was not received within three months, the ATTS-BR evaluation process would proceed to subsequent stages.

### Evaluation by experts

The final translated version of the ATTS was evaluated by an Expert Committee composed of professionals from various health areas with a focus on mental health, experts in instrument validation and specialists in suicidology. Multidisciplinarity and fluency in English and Brazilian Portuguese were used as selection criteria^(24)^. Consultations carried out on the Lattes and LinkedIn platforms and recommendations from the research group’s network of contacts were used to reach the professionals who met the established criteria.

The contact and invitation were made via email, and those who agreed to collaborate with the study received a link to access an electronic form on REDCap (Research Electronic Data Capture)^(25,26)^ with the Informed Consent form (IC Experts version), followed by a sociodemographic questionnaire, the consensual version of the ATTS translated into Portuguese, and the questionnaire to evaluate this version. After all the completed forms were returned, the experts were invited to a synchronous virtual meeting to improve the items of the instrument considered as unsatisfactory.

Initially, 27 invitations were sent via email to the experts that had been previously selected through CV analysis, and only 11 responded to the invitation accepting to participate. After confirmation, three possible dates and times for the synchronous virtual meeting were sent, and nine experts agreed to participate.

The sociodemographic questionnaire prepared for the study contained questions related to gender, age, city and state of residence, religious beliefs, marital status, highest degree, length of experience in the mental health field, and current occupation. The following equivalences were considered as criteria in the evaluation of the ATTS: semantic (related to the meaning of words); idiomatic (colloquial expressions and specificities of languages that may be difficult to translate); experiential (cross-cultural appropriateness of the situations described in the instrument); and conceptual (cross-cultural validity of the concept studied)^(24)^. The experts were instructed that the item should be assessed as appropriate when the four aforementioned criteria were met. If the inappropriate option was selected, response options would be provided in the questionnaire to indicate the inappropriate equivalence.

Sociodemographic data were subjected to descriptive analysis. In the assessment of the ATTS, the Content Validity Index (CVI), which represents the proportion or percentage of experts in agreement on the instrument item, was calculated for each item. All items that met the minimum acceptable criterion (80.0%) of agreement among the experts were analyzed by them in a synchronous meeting^(27)^. The final version of the ATTS in Brazilian Portuguese (ATTS-BR) was obtained at the end of the meeting.

### Target audience assessment

The ATTS-BR was submitted to pre-testing, a stage in which 38 individuals were invited to participate. As three refused, the final sample consisted of 35 individuals from the general population, a number compatible with the adopted reference, which recommends 30 to 40 participants^(21)^.

Potential participants were approached by the principal investigator in high-traffic locations (public places such as squares and bus terminals, in order to ensure heterogeneous participation of people eligible for the study) and informed about the purpose of the study. After consent and signature of the IC form, participants completed a sociodemographic questionnaire, the ATTS-BR and a form with assessment of the ATTS-BR based on the work of Deon et al.^(28)^ and the general guidelines of the study by Beaton et al.^(21)^. Participants aged between 18 and 65 years, able to read and understand the text without assistance, fluent in Brazilian Portuguese and available for collection were included in the study.

The sociodemographic questionnaire included questions on age, gender, city and state of residence, educational level, and on factors associated with suicide-related attitudes described in the scientific literature^(8)^. The ATTS-BR evaluation questionnaire contained questions about the ease of understanding of the instrument and each item, the importance of each item, response options, and suggestions for changes.

At the end of the questionnaires, an invitation to a virtual meeting (optional) with participants of the pre-test was made available. Participants interested in collaborating in the discussion were asked to provide their preferred contact method for receiving the link to the virtual meeting. In total, seven participants provided their contact method for accessing the invitation to the virtual meeting. Considering the results obtained from analysis of data with the target audience, a meeting after the pre-test was not necessary, nor was a new round of review by the Expert Committee.

The data collected were coded and double-entered into Microsoft Excel spreadsheets, then compared, corrected, and descriptive analysis was performed. The IBM^®^ SPSS^®^ Statistics version 25 was used in statistical analyzes.

## RESULTS

The ATTS instrument underwent two independent translations (versions T1 and T2). A total of 12 items were translated with the same spelling and the rest were reviewed in a meeting with the translators to create a consensual version in Brazilian Portuguese (T1+2). In the back-translation of the consensual version (T1+2), 10 items were translated identically by both translators. The remaining items were analyzed in a synchronous virtual meeting with the translators to establish the consensual version of the back-translation (R1+2). The back-translation was sent to the authors of the original scale, who did not return the contact with suggestions for changes.

The translators of each stage (translation and back-translation) were instructed to maintain the original meanings of the instrument items in order to ensure that the new version was faithful to the original instrument, even with contextual adaptations, specificities of the new language and adherence to recommendations on suicide-related safe communication. The analysis of semantic, idiomatic, experiential and conceptual equivalences was carried out by the Expert Committee in order to ensure the practical applicability of the terms present in the ATTS.

Thus, all items of the consensual version of the ATTS in Portuguese (T1+2) were evaluated by the Expert Committee. Most experts were female (77.7%), had religious beliefs (55.5%), had a partner (66.6%), lived in the state of São Paulo (66.7%), had at least a doctorate degree (55.5%) and had worked for 0-10 years in the mental health area (44.5%).

Most of the instrument items reached or exceeded the minimum CVI value (80.0%). However, items 3, 7, 9, 10, 12, 17, 30, 36, 38 and 40 did not reach the established minimum agreement criteria and were modified in relation to the wording during the meeting held with the experts. Items 6, 11 and 39 were revised again under guidance of the translators who participated in the first stage.

### Evaluation of the ATTS-BR by the target audience

The final version prepared after the experts’ evaluation was called ATTS-BR, and underwent evaluation by the target audience. This stage of the study included the participation of 35 adults with average age of 36.3 years (SD=12.4; Median: 34.0 years), cisgender women (67.6%), with religious beliefs (74.3%), with a partner (61.8%) and complete higher education (29.4%). Regarding previous contact with suicidal behavior, the majority reported not having read materials on the subject (55.9%), denied knowing anyone close to them who had died by suicide (68.6%) or had attempted suicide (57.1%).

Most participants evaluated the ATTS as good (88.6%), containing items that were easy to understand (88.6%) and important (94.3%). Of the total number of participants, 80.0% denied having difficulty understanding the response options, 91.4% did not offer any suggestions for improving the ATTS-BR, and 91.2% considered there was no need to make changes to the instrument. The suggestions for improving the instrument given by 0.6% of participants were carefully analyzed. Since there were no specific suggestions for improving the instrument, it was kept unchanged.

The assessment of the importance of the ATTS-BR items indicated that all items were considered important by most respondents. The items considered as more important were: item 37 “*O suicídio pode ser prevenido*”, considered important by 100.0% of participants; item 23 “*A maioria das pessoas evita falar sobre suicídio*” and item 26 “*Uma tentativa de suicídio é essencialmente um grito de socorro*”, classified as important by 97.1% of participants. Items 1 “*Sempre é possível ajudar uma pessoa com pensamentos suicidas*”, 3 “*A morte por suicídio é uma das piores coisas que pode acontecer em uma família*” and 9 “*É dever de todos tentar impedir alguém de morrer por suicídio*” were considered important by 94.3% of participants.

Participants evaluated each item by stating whether it was difficult to understand or not. In all items, the majority denied having any difficulties in understanding. Item 3 was the statement in which the most people (20.7%) had some difficulty understanding.

The ATTS statements that presented the highest agreement scores were: item 37 “*O suicídio pode ser prevenido*” (91.4%); item 2 “*A morte por suicídio é uma das piores coisas que pode acontecer em uma família*” (88.5%); item 23 “*A maioria das pessoas evita falar sobre suicídio*” (85.7%); item 26 “*Uma tentativa de suicídio é essencialmente um grito de socorro*” (85.7%); item 1 “*Sempre é possível ajudar uma pessoa com pensamentos suicidas*” (82.8%); and item 9 “*É dever de todos tentar impedir alguém de morrer por suicídio*” (82.8%) (Table 1).

**Table 1:** Frequency of scores of participants’ responses regarding agreement and/or disagreement with the statements described in the items of the Suicide Attitudes questionnaire by the target audience (N = 35), Ribeirão Preto, São Paulo, Brazil, 2024

Itens			%		
	Concordo Totalmente	Concordo	Indeciso	Discordo	Discordo Totalmente
1. Sempre é possível ajudar uma pessoa com pensamentos suicidas.	37.1	45.7	5.7	8.6	2.9
2. O suicídio nunca pode ser justificado.	11.4	11.4	25.7	40.0	11.4
3. A morte por suicídio é uma das piores coisas que pode acontecer em uma família.	57.1	31.4	2.9	8.6	-
4. A maioria das tentativas de suicídio são ações impulsivas.	5.7	34.3	11.4	42.9	5.7
5. O suicídio é um meio aceitável de acabar com uma doença incurável.	-	5.7	17.1	42.9	34.3
6. Uma vez que uma pessoa tenha decidido morrer por suicídio ninguém consegue impedi-la.	-	8.6	8.6	68.6	14.3
7. Muitas tentativas de suicídio são por vingança ou para punir outra pessoa.	-	11.4	11.4	45.7	31.4
8. Pessoas que morrem por suicídio geralmente têm transtornos mentais.	11.4	34.3	5.7	42.9	5.7
9. É dever de todos tentar impedir alguém de morrer por suicídio.	25.7	57.1	8.6	8.6	-
10. Quando uma pessoa morre por suicídio é algo que ela considerou por muito tempo.	11.4	42.9	22.9	22.9	-
11. Perguntar sobre suicídio pode provocar pensamentos suicidas na pessoa.	-	11.4	17.1	60.0	11.4
12. Pessoas que ameaçam suicídio raramente se suicidam.	-	8.6	20.0	57.1	14.3
13. O suicídio é um assunto sobre o qual não se deve falar.	-	5.7	5.7	37.1	51.4
14. A solidão pode ser para mim um motivo para tirar minha vida.	2.9	14.3	2.9	45.7	34.3
15. Quase todo mundo, em algum momento, já pensou em suicídio	5.7	25.7	14.3	42.9	11.4
16. Pode haver situações em que a única solução razoável seja o suicídio.	-	8.6	5.7	51.4	34.3
17. Eu poderia dizer que me mataria sem ter a intenção de realizar o ato.	5.7	31.4	17.1	25.7	20.0
18. Às vezes o suicídio pode ser um alívio para os envolvidos.	-	28.6	14.3	34.3	22.9
19. Suicídios entre jovens são mais difíceis de entender, pois eles teriam muita vida pela frente.	14.3	45.7	8.6	20.0	11.4
20. Eu consideraria a possibilidade de tirar minha vida se eu sofresse de uma doença grave e incurável.	-	14.3	20.0	34.3	31.4
21. Uma vez que a pessoa teve pensamentos suicidas, nunca deixará de pensar sobre isso.	2.9	14.3	25.7	48.6	8.6
22. O suicídio acontece sem aviso prévio.	11.4	17.1	2.9	60.0	8.6
23. A maioria das pessoas evita falar sobre suicídio.	11.4	74.3	8.6	5.7	-
24. Se uma pessoa quiser se matar, é problema dela e não devemos interferir.	-	5.7	-	37.1	57.1
25. A solidão é o principal motivo que leva as pessoas ao suicídio.	8.6	28.6	17.1	42.9	2.9
26. Uma tentativa de suicídio é essencialmente um grito de socorro.	34.3	51.4	2.9	11.4	-
27. No geral, não entendo como as pessoas podem tirar a própria vida.	25.7	31.4	14.3	25.7	2.9
28. Normalmente, os familiares não têm ideia do que está acontecendo quando uma pessoa está pensando em suicídio.	14.3	54.3	8.6	20.0	2.9
29. Se uma pessoa está sofrendo de uma doença grave, incurável, e expressa o desejo de morrer, deve obter ajuda para isso.	14.3	25.7	17.1	25.7	17.1
30. Me sinto preparado para fazer contato e ajudar uma pessoa que esteja em crise suicida.	5.7	37.1%	14.3	31.4	11.4
31. Qualquer pessoa pode morrer por suicídio.	8.6	40.0	25.7	17.1	8.6
32. É compreensível que pessoas que sofrem de uma doença grave e incurável tirem a própria vida.	2.9	37.1	14.3	34.3	11.4
33. Pessoas que falam sobre suicídio não tiram a própria vida.	-	11.4	25.7	42.9	20.0
34. As pessoas têm o direito de tirar suas próprias vidas.	-	20.0	14.3	31.4	34.3
35. A maioria das tentativas de suicídio é causada por conflitos com uma pessoa próxima.	5.7	20.0	22.9	51.4	-
36. Eu gostaria de obter ajuda para morrer por suicídio se eu tivesse uma doença grave e incurável.	2.9	22.9	17.1	22.9	34.3
37. O suicídio pode ser prevenido.	40.0	51.4	5.7	2.9	-

The items with the highest scores of disagreement with the statement were: item 24 “*Se uma pessoa quiser se matar, é problema dela e não devemos interferir*” (94.2%); item 13 “*O suicídio é um assunto sobre o qual não se deve falar*” (88.5%); item 16 “*Pode haver situações em que a única solução razoável seja o suicídio*” (85.7%); item 6 “*Uma vez que uma pessoa tenha decidido morrer por suicídio ninguém consegue impedi-la*” (82.9%); and item 14 “*A solidão pode ser para mim um motivo para tirar minha vida*” (80.0%) (Table 1).

The statements that presented the highest indecision scores obtained a value of 25.7% of responses: item 2 “*O suicídio nunca pode ser justificado*”; item 21 “*Uma vez que a pessoa teve pensamentos suicidas, nunca deixará de pensar sobre isso*”; and item 31 “*Qualquer pessoa pode morrer por suicídio*” (Table 1).

## DISCUSSION

The stages of this study were planned and conducted in accordance with internationally recognized recommendations widely used in the national and international context for the design of translation and cross-cultural adaptation studies^(21,24)^. Items evaluated as appropriate by at least 80.0% of the experts were considered appropriate^(27)^. The definition of acceptable CVI values provides greater objectivity in the analysis of the items^(27)^.

Most items obtained CVI higher than the established criterion and the items that did not reach this value were improved in a synchronous meeting with the experts, allowing the final version of the ATTS-BR to be carefully constructed. The care taken in the initial stages of translation and adaptation allowed the construction of an instrument evaluated by the target audience as good, easy to understand, important, with comprehensible response options and no need for changes or improvements.

Some participants reported contact with people who attempted suicide (42.9%) or died by suicide (31.4%). These findings may be related to the taboo associated with the topic, which may cause people not to report suicidal thoughts or behaviors in family members to strangers with the aim of protecting family members from possible stigma and avoiding exposure to the topic^(20,29)^.

The items with the highest disagreement scores were related to unfavorable attitudes toward prevention, the person’s motivation for attempting suicide, and silence regarding the topic. The statements with the highest agreement scores were related to favorable attitudes toward prevention and beliefs that suicide is a painful and poorly discussed subject. Item 37, for example, assessed the belief in the possibility of prevention, and 91.4% of participants agreed that suicide can be prevented. Studies show that women have more favorable attitudes toward prevention than men^(20)^. In another study in which the ATTS was also used to assess the attitudes of mental health professionals working in care, high agreement with the possibility of suicide prevention was found^(8)^.

Some questions on the ATTS are directly related to the acceptability of suicide, an attitudinal dimension related to less condemnatory behavior, but also to a greater propensity for suicidal behavior and less investment or credibility in prevention actions^(30,31)^. A study conducted in South Korea demonstrated that more permissive attitudes were associated with the frequency of suicidal behavior, plans and ideations, as well as with the risk of suicide attempts^(30)^. Issues of gender, religion and political views are other predictors associated with acceptability^(7)^.

The acceptability of suicide is also assessed in specific situations, such as serious and incurable diseases. In the present study, a minority of participants agreed that suicide is an acceptable method in situations of incurable disease. However, in the literature, 61.0% of participants agreed that suicide is an acceptable method in the case of incurable diseases, since death in these conditions can be considered inevitable, while it can be prevented in other situations and/or circumstances^(7)^.

Regarding the association between mental disorders and suicide addressed in item 8, 45.7% agreed and 48.6% disagreed with this relationship. Not all people who die by suicide have some mental disorder and most people with mental disorders do not die by suicide^(8)^. Other studies reinforce that professionals working in the mental health area tend to consider mental disorders as the main cause associated with suicide^(8)^ and emphasize their agreement on the need for further study on the subject^(32)^.

The perspective of suicide as an act of impulsivity addressed in item 4 of the ATTS contrasts with the belief of 48.6% of participants. Impulsivity in isolation does not fully explain suicidal behavior, but it can be configured as a risk factor for suicide attempts, since it can play an important role in the transition from thought to action and mediate the progression of ideation to suicide attempts^(1)^.

The ATTS also assesses beliefs about the association between loneliness and suicide. It was observed that 37.2% of participants agreed that loneliness is the main reason that leads people to commit suicide (item 25). Different theoretical models address interactional aspects as some of the various factors associated with suicide that can manifest as protection (belonging, connection, satisfactory relationships) or as risk factors (loneliness, isolation, discrimination, violence)^(33,34)^. Although these factors cannot be understood in isolation in suicide, the literature points to the importance of strengthening the support network as a strategy for suicide prevention^(30)^.

In item 23, 85.7% of participants agreed that “*a maioria das pessoas evita falar sobre o suicídio*” (most people avoid talking about suicide) and 42.8% disagreed with the statement in item 30 “*me sinto preparado para fazer contato e ajudar uma pessoa que esteja em crise suicida*” (I feel prepared to make contact and help a person who is in suicidal crisis). These analyzes highlight the importance of discussions involving the topic with the aim to perform health education activities focused on the population. Suicide is a commonly avoided topic, which people feel insecure or uncomfortable to talk about^(35)^. This silence or avoidance can limit the possibilities of seeking help and implementing preventive and care actions. Talking about the subject assertively can help identify underlying problems and make it possible to overcome introspection during the dialogue^(8)^.

In the current context, beliefs that suicide can be caused by talking about the subject persist. In this study, the low agreement with item 11, “*perguntar sobre suicídio pode provocar pensamentos suicidas na pessoa”* (asking about suicide can trigger suicidal thoughts in the person), may indicate a process of valuing the importance of talking about suicide. In this scenario, it becomes important to disseminate knowledge related to safe communication about suicide^(36)^.

The possibility of perceiving signs indicating the risk of suicide requires careful and thoughtful consideration. In this study, the percentage of agreement with item 22 (28.5%) was close to the findings of Hjelmeland and Knizek^(37)^, in which 29% of participants agreed with the statement that “*suicídio ocorre sem aviso prévio*” (suicide occurs without warning). The authors address the importance of not underestimating the signs indicating risk and taking them seriously^(37)^. However, suicidal behavior is not predictable and some signs of risk may be nonspecific^(38)^.

A study conducted nationwide showed an increase in reports of self-harm in all regions of Brazil^(39)^. These rates were more frequent among women, while deaths by suicide, which also increased in all Brazilian regions, were more frequent in men aged 25 to 59 years, which includes a large part of the sample in this study^(39)^. Suicide rates showed an average increase of 3.70% per year between 2011 and 2022, with a rate of 7.3 per 100,000 inhabitants in the last year^(39)^.

The literature has shown that content and interactions mediated by technologies and the internet seem to influence attitudes towards suicide, since people are exposed to a variety of information and ideas, and can interact differently in relation to this subject^(7)^. The characteristics of the relationships between virtual communications and attitudes related to suicidal behavior can be further explored in Brazil after the evaluation of the psychometric characteristics of the ATTS-BR.

The cross-cultural adaptation of the ATTS questionnaire is expected to present satisfactory psychometric values for its application and offer another possible instrument for assessing attitudes toward suicide in the Brazilian context. In addition, it will allow the analysis of the influence of sociodemographic factors such as gender, culture, and age on attitudes toward suicide in the national context.

### Study limitations

Convenience sampling in the pre-test stands out as a limitation since it does not allow generalization of data if regional specificities and intersectional aspects are considered. The need for studies that validate the psychometric properties of the instrument is also highlighted.

### Contributions to the area of nursing, health, or public policy

The instrument has the potential to assess the effect of interventions related to mental health literacy, from the provision of an instrument that can be validated in other ways and contribute to discussions, educational actions, interventions and policies that consider attitudes related to suicide in their planning and scope of actions.

## CONCLUSIONS

The Attitudes Towards Suicide questionnaire was translated and adapted to the Brazilian context using processes recommended in widely used international references on the method. The instrument was translated, back-translated, evaluated by experts on the subject, and semantic evaluation by the target population of the study was performed as well. Most of the target audience evaluated the final version of the questionnaire positively, considering the items important, easy to understand and with no need for modifications. The instrument allowed the analysis of factors that may influence attitudes towards suicide in the national context in order to support better interventions and strategic actions focused on suicide prevention.

## Data Availability

The research data are available within the article.
