# Supplementary material for: Attitudes Towards Suicide Questionnaire (ATTS): translation and adaptation to the Brazilian context
Source: Rev Bras Enferm. 2025 Sep 5;78(4):e20240575. doi: 10.1590/0034-7167-2024-0575 (PMC12419753; doi:10.1590/0034-7167-2024-0575)
Supplement: Translation

# Translation: Questionário de Atitudes Frente ao Suicídio (ATTS): tradução e adaptação para o contexto brasileiro

**DOI:** 10.1590/0034-7167-2024-0575pt

**Authors:** Bruna Marques Chiarelo, Laysa Fernanda Silva Pedrollo, Micael Doria de Andrade, Ana Carolina Guidorizzi Zanetti, Kelly Graziani Giacchero Vedana

**Affiliations:** I Universidade de São Paulo. Ribeirão Preto, São Paulo, Brasil

**Keywords:** Atitude, Suicídio, Tradução, Estudo Metodológico, Prevenção do Suicídio.

## Abstract

**Objetivos:**

traduzir e adaptar o questionário de Atitudes Frente ao Suicídio para o contexto brasileiro.

**Métodos:**

estudo metodológico, transversal. O instrumento em sua versão original inglesa foi traduzido para o português brasileiro, posteriormente submetido a uma retrotradução para a língua original e encaminhado para os autores originais. Em seguida, foi apreciado por um Comitê de Especialistas composto por nove integrantes e avaliado através de um pré-teste com 35 participantes da população geral de 18 a 65 anos. Os dados foram codificados e analisados através do *software* estatístico.

**Resultados:**

a maioria dos participantes avaliou o questionário como bom (88,6%); com itens importantes (94,3%); fáceis de entender (88,6%) e sem necessidade de mudanças (91,2%).

**Conclusões:**

o instrumento permite a análise de fatores que podem influenciar as atitudes frente ao suicídio no contexto nacional. Essas informações poderão subsidiar melhores intervenções e ações estratégicas de prevenção do suicídio.

## INTRODUÇÃO

O suicídio é definido como a morte autoprovocada, com evidências de que a pessoa tinha intenção de morrer^(1)^. O fenômeno é complexo, multifatorial e um importante problema de saúde pública^(2)^. Estima-se que 703.000 pessoas morrem anualmente por suicídio^(3)^. No Brasil, a taxa de suicídio é de 6.4 para cada 100.000 habitantes. De acordo com as estimativas globais, foram registrados 14.540 suicídios no ano de 2019^(3)^.

As atitudes relacionadas ao suicídio podem interferir em comportamentos de pedido e oferta de ajuda^(4)^. Evidências disponíveis mostraram que essas atitudes podem variar de acordo com características sociodemográficas como gênero, etnia, cultura, experiências com o suicídio de membros da família e pessoas significativas, expressões do comportamento suicida ao longo da vida, idade^(5-7)^ e crenças religiosas^(8,9)^. Contudo, a associação entre a formação profissional relacionada ao suicídio e melhores atitudes é frequentemente identificada^(4)^.

A avaliação dessas atitudes pode auxiliar na elaboração de políticas e estratégias voltadas à prevenção^(10)^ e na aplicação de intervenções coerentes ao contexto e condições singulares vivenciadas por grupos com necessidades e recursos específicos, possibilitando um planejamento mais assertivo, diverso, inclusivo e equitativo^(6)^. Os diversos fatores que interferem nas atitudes evidenciam os desafios para mensurar o construto de forma criteriosa e abrangente^(11,12)^.

O Questionário sobre Atitudes Frente ao Comportamento Suicida (QACS) foi o único instrumento disponível no contexto brasileiro com esse enfoque. Contudo, seu uso é restrito aos profissionais da saúde e o instrumento possui limitações relacionadas à avaliação restrita a poucas dimensões das atitudes frente ao suicídio, pouca praticidade para obtenção dos escores (conversão manual da escala visual em pontuação) e inutilização de alguns itens da escala para o cálculo dos escores dos fatores^(13)^.

No contexto internacional, destaca-se o questionário *Attitudes Towards Suicide (ATTS)* por ser um instrumento amplamente utilizado e considerar os aspectos multidimensionais das atitudes relacionadas ao suicídio. Trata-se de um instrumento elaborado por Renberg e Jacobsson^(14)^ e baseado no Suicide Opinion Questionnaire (SOQ) e Suicide Attitude Questionnaire (SUIATT)^(15-17)^. A sua ampla utilização se deve à quantidade de itens, aos dez fatores avaliados, às dimensões envolvidas e às características psicométricas satisfatórias apresentadas pelo instrumento^(17)^.

O ATTS foi adaptado e validado para diferentes países como Suécia, Noruega, Rússia^(6)^, Índia^(5)^, Malásia^(18)^, Japão^(19)^, Coreia do Sul^(10)^, Polônia^(17)^, Irã^(20)^ e tem sido utilizado como um importante instrumento para avaliar as atitudes relacionadas ao suicídio em diversas populações. No entanto, não há evidências da adaptação do ATTS para o contexto brasileiro, o que reforça a possibilidade de disponibilizar o instrumento para avaliação de atitudes frente ao suicídio no país.

## OBJETIVOS

Traduzir e adaptar o questionário de Atitudes Frente ao Suicídio para o contexto brasileiro.

## MÉTODOS

### Tradução e Retrotradução

Estudo metodológico de tradução e adaptação transcultural. O desenvolvimento da pesquisa foi aprovado pelo Comitê de Ética e Pesquisa da Escola de Enfermagem de Ribeirão Preto da Universidade de São Paulo (EERP/USP). A autorização dos autores originais do instrumento foi obtida previamente para realização do estudo.

A versão do ATTS adaptada e validada foi a de 34 itens. Essa versão apresenta uma escala tipo *Likert* de 1 a 5 pontos, que varia entre “*Strongly Disagree*” a “*Strongly Agree*”. Quanto maior a concordância, maior a pontuação, com exceção aos itens 7 e 9, que são avaliados de forma invertida. O instrumento avalia 10 fatores, sendo estes: *Suicide as a right* (Fator 1)*, Incomprehensibility* (Fator 2)*, Noncommunication* (Fator 3)*, Preventability* (Fator 4)*, Tabooing* (Fator 5)*, Normal-common* (Fator 6)*, Suicidal process* (Fator 7)*, Relation-caused* (Fator 8)*, Preparedness to prevent* (Fator 9)*, Resignation* (Fator 10), com a consistência interna dos itens variando de 0,38 a 0,86. O instrumento completo apresentou consistência interna de 0,60^(14)^.

O processo de adaptação transcultural do estudo seguiu as recomendações da literatura^(21,22)^. Para obter a primeira versão consensual em português, foram realizadas duas traduções do inglês para a língua portuguesa de forma independente por dois tradutores bilíngues, familiarizados com as duas culturas e que tinham como língua materna o português do Brasil. Os mesmos foram orientados quanto à necessidade do uso de uma linguagem clara e simples^(21)^. Em uma reunião com os tradutores, as duas versões produzidas foram comparadas para elaborar uma versão consensual em português. Os tradutores receberam orientações sobre a comunicação segura relacionada ao suicídio para que fossem evitadas expressões estigmatizantes ou contraindicadas, tais como “cometer suicídio”, “suicídio completo”, entre outras^(23)^.

Com a finalidade de avaliar inconsistências, a equivalência conceitual e a qualidade da tradução, a versão consensual em português foi submetida à retrotradução (para língua inglesa), por dois tradutores nativos, de forma independente, bilíngues e que tinham o inglês como língua materna^(21)^. Em uma reunião com os tradutores, as duas versões da retrotradução foram comparadas para construção de uma versão final consensual da retrotradução em inglês. A versão construída foi enviada aos autores originais do estudo para apreciação em relação a possíveis incongruências ou inconsistências, considerando a proposta original do ATTS e sua finalidade. Foram enviados três *e-mails* aos autores originais do ATTS com a solicitação de que, no prazo de três meses, indicassem a necessidade de alterações. Os autores foram informados que, na ausência de resposta dentro deste período, o processo de avaliação do ATTS-BR seguiria para as etapas subsequentes.

### Avaliação por especialistas

A versão final traduzida do ATTS foi então submetida à avaliação por um Comitê de Especialistas composto por profissionais de diversas áreas da saúde com enfoque na saúde mental, com expertise na validação de instrumentos e especialistas em suicidologia. A multidisciplinaridade e a fluência nas línguas inglesa e portuguesa do Brasil foram utilizadas como critério de seleção^(24)^. Consultas realizadas nas plataformas Lattes e Linkedin, bem como indicações da rede de contatos do grupo de pesquisa foram utilizados para alcançar os profissionais que atendessem aos critérios estabelecidos.

O contato e o convite ocorreram por e-mail e os convidados que aceitaram colaborar com a pesquisa receberam um link de acesso a um formulário eletrônico no REDCap (*Research Electronic Data Capture*)^(25,26)^, com Termo de Consentimento Livre e Esclarecido (TCLE versão Especialistas), seguido por um questionário sociodemográfico, a versão consensual do ATTS traduzida para o português e o questionário para avaliação dessa versão. Após recebimento de todos os formulários preenchidos, os especialistas foram convidados para uma reunião virtual síncrona, com a finalidade de aprimorar os itens do instrumento considerados insatisfatórios.

Inicialmente, 27 convites foram enviados via *e-mail* para especialistas selecionados previamente nas análises de currículo e apenas 11 convidados retornaram ao convite com o aceite de participação. Após a confirmação, foram enviadas três possibilidades de data e horário para realização de reunião virtual síncrona e nove especialistas aceitaram participar do encontro.

O questionário sociodemográfico elaborado para o estudo continha questões relacionadas ao gênero, idade, cidade e estado de residência, crença religiosa, estado civil, titulação máxima, tempo de atuação na área de saúde mental e ocupação atual. As seguintes equivalências foram consideradas como critérios na avaliação do ATTS: semântica (relacionada ao significado das palavras); idiomática (expressões coloquiais e especificidades dos idiomas que podem ser difíceis de traduzir); experiencial (adequação transcultural das situações descritas no instrumento); e conceitual (validade transcultural do conceito estudado)^(24)^. Os especialistas foram instruídos de que o item deveria ser avaliado como adequado se contemplasse os quatro critérios supracitados. Caso a opção inadequada fosse selecionada, o questionário disponibilizaria opções-respostas para indicar a equivalência inadequada.

Os dados sociodemográficos foram submetidos à análise descritiva. Na avaliação do ATTS, o Índice de Validade de Conteúdo (IVC), que representa a proporção ou porcentagem de especialistas em concordância sobre o item do instrumento, foi calculado para cada item. Todos os itens que atingiram o critério mínimo aceitável (80,0%) de concordância entre os especialistas foram analisados em uma reunião síncrona^(27)^. A versão final do ATTS na língua portuguesa do Brasil (ATTS-BR) foi obtida ao final da reunião.

### Avaliação pelo público-alvo

O ATTS-BR foi submetido ao pré-teste, etapa da qual 38 indivíduos foram convidados a participar. Como três recusaram, a amostra final foi composta por 35 indivíduos da população geral, quantidade compatível com o referencial adotado, que recomenda de 30 a 40 participantes^(21)^.

Os potenciais participantes foram abordados pela pesquisadora responsável em locais de grande circulação (locais públicos como praças e terminais rodoviários, no intuito de garantir uma participação heterogênea de pessoas elegíveis para o estudo) e informados sobre o objetivo da pesquisa. Após consentimento informado e assinatura do TCLE, os participantes preencheram questionário sociodemográfico, o ATTS-BR e um formulário com a avaliação do ATTS-BR fundamentado no trabalho de Deon et al.^(28)^ e nas orientações gerais do estudo de Beaton et al.^(21)^. Participantes com idades entre 18 e 65 anos, capacidade de ler e compreender texto sem auxílio, fluência na língua portuguesa do Brasil e disponibilidade na coleta foram incluídos no estudo.

O questionário sociodemográfico abrangeu questões sobre idade, gênero, cidade e estado de residência, escolaridade e questões sobre fatores associados às atitudes relacionadas ao suicídio descritas na literatura científica^(8)^. O questionário de avaliação do ATTS-BR conteve questões sobre a facilidade de compreensão do instrumento e de cada item, a importância de cada item, opções de respostas e sugestões de mudanças.

Ao final dos questionários, foi disponibilizado um convite para uma reunião virtual (opcional) com os participantes do pré-teste. Aos interessados em colaborar na discussão, foi solicitado informar a via de contato de preferência para o envio do link da reunião virtual. Ao todo, sete participantes informaram a forma de contato para acesso ao convite da reunião virtual. Considerando os resultados obtidos pelas análises dos dados com o público-alvo, a reunião após o pré-teste não foi necessária, nem nova rodada de revisão pelo Comitê de especialistas.

Os dados coletados foram codificados e duplamente digitados em planilhas do *Microsoft Excel*, em seguida comparados, corrigidos e submetidos à análise descritiva. O *software* IBM^®^ SPSS^®^ Statistics, versão 25 foi utilizado nas análises estatísticas.

## RESULTADOS

O instrumento ATTS foi submetido a duas traduções independentes (versões T1 e T2). Um total de 12 itens foi traduzido com a mesma grafia e os demais foram revisados em uma reunião com os tradutores para construção de uma versão consensual em português do Brasil (T1+2). Na retrotradução da versão consensual (T1+2), 10 itens foram traduzidos de forma idêntica pelos dois tradutores. Os demais itens foram analisados em um encontro virtual síncrono com os tradutores para estabelecer a versão consensual da retrotradução (R1+2). A retrotradução foi enviada aos autores da escala original, que não retornaram o contato com apontamentos para alterações.

Os tradutores de cada uma das etapas (tradução e retrotradução) foram instruídos a manter os significados originais dos itens do instrumento, com o intuito de possibilitar que a nova versão fosse fidedigna ao instrumento original, mesmo com adaptações contextuais, especificidades da nova língua e adesão às recomendações sobre comunicação segura relacionada ao suicídio. A análise das equivalências semântica, idiomática, experiencial e conceitual foi realizada pelo Comitê de Especialistas com a finalidade de garantir a aplicabilidade prática relacionada aos termos presentes no ATTS.

Assim, todos os itens da versão consensual do ATTS em português (T1+2) foram submetidos à avaliação pelo Comitê de Especialistas. A maioria dos especialistas era do gênero feminino (77,7%), referiu possuir crença religiosa (55,5%), ter companheiro (66,6%), morava no estado de São Paulo (66,7%), tinha ao menos a titulação de doutorado (55,5%) e atuava de zero a 10 anos na área de saúde mental (44,5%).

A maioria dos itens do instrumento atingiu ou superou o valor mínimo de IVC (80,0%). No entanto, os itens 3, 7, 9, 10, 12, 17, 30, 36 e 38 e 40 não atingiram os critérios de concordância mínima estabelecidos e foram modificados em relação à redação durante a reunião realizada com os especialistas. Os itens 6,11 e 39 foram novamente revisados por orientação dos tradutores que participaram da primeira etapa.

### Avaliação do ATTS-BR pelo público-alvo

A versão final elaborada após a avaliação dos especialistas foi denominada ATTS-BR, e submetida à apreciação pelo público-alvo. Esta etapa do estudo contou com a participação de 35 adultos com média de idade de 36,3 anos (DP=12,4; Mediana: 34,0 anos), mulheres cisgênero (67,6%), com crença religiosa (74,3%), com companheiro (61,8%) e ensino superior completo (29,4%). Com relação ao contato prévio com o comportamento suicida, a maioria referiu não ter lido materiais sobre o tema (55,9%), negou conhecer alguém próximo que tenha morrido por suicídio (68,6%) ou tido tentativas de suicídio (57,1%).

A maioria dos participantes avaliou o ATTS como bom (88,6%) contendo itens fáceis de entender (88,6%) e importantes (94,3%). Do total de participantes, 80,0% negaram ter dificuldades para compreender as opções de resposta, 91,4% não ofereceram sugestões para o aprimoramento do ATTS-BR e 91,2% consideraram que não era necessário realizar mudanças no instrumento. As sugestões informadas por 0,6% dos participantes para o aprimoramento do instrumento foram cuidadosamente analisadas. Como não houve sugestões específicas para aprimorar o instrumento, ele foi mantido sem alterações.

A avaliação sobre a importância dos itens do ATTS-BR apontou que todos os itens foram considerados importantes pela maioria dos respondentes. Os itens considerados mais importantes foram: o 37 “O suicídio pode ser prevenido”, considerado importante por 100,0% dos participantes; e o 23 “A maioria das pessoas evita falar sobre suicídio” e o 26 “Uma tentativa de suicídio é essencialmente um grito de socorro”, classificados como importantes por 97,1% dos participantes. Os itens 1 “Sempre é possível ajudar uma pessoa com pensamentos suicidas”, 3 “A morte por suicídio é uma das piores coisas que pode acontecer em uma família” e 9 “É dever de todos tentar impedir alguém de morrer por suicídio”, foram considerados importantes para 94,3% dos participantes.

Os participantes avaliaram cada item declarando se ele foi difícil de entender ou não. Em todos os itens, a maioria negou dificuldades de compreensão. O item 3 foi a afirmativa com mais pessoas (20,7%) com alguma dificuldade de compreensão.

As afirmações do ATTS que apresentaram maiores escores de concordância foram: o item 37 “O suicídio pode ser prevenido” (91,4%); item 2 “A morte por suicídio é uma das piores coisas que pode acontecer em uma família” (88,5%); item 23 “A maioria das pessoas evita falar sobre suicídio” (85,7%); item 26 “Uma tentativa de suicídio é essencialmente um grito de socorro” (85,7%); item 1 “Sempre é possível ajudar uma pessoa com pensamentos suicidas” (82,8%); e o item 9 “É dever de todos tentar impedir alguém de morrer por suicídio” (82,8%) (Tabela 1).

**Tabela 1:** Frequência de escores das respostas dos participantes sobre a concordância e/ou discordância com as afirmações descritas nos itens do Questionário de Atitudes Frente ao Suicídio pelo público-alvo (N = 35), Ribeirão Preto, São Paulo, Brasil, 2024

Itens			%		
	Concordo Totalmente	Concordo	Indeciso	Discordo	Discordo Totalmente
1. Sempre é possível ajudar uma pessoa com pensamentos suicidas.	37,1	45,7	5,7	8,6	2,9
2. O suicídio nunca pode ser justificado.	11,4	11,4	25,7	40,0	11,4
3. A morte por suicídio é uma das piores coisas que pode acontecer em uma família.	57,1	31,4	2,9	8,6	-
4. A maioria das tentativas de suicídio são ações impulsivas.	5,7	34,3	11,4	42,9	5,7
5. O suicídio é um meio aceitável de acabar com uma doença incurável.	-	5,7	17,1	42,9	34,3
6. Uma vez que uma pessoa tenha decidido morrer por suicídio ninguém consegue impedi-la.	-	8,6	8,6	68,6	14,3
7. Muitas tentativas de suicídio são por vingança ou para punir outra pessoa.	-	11,4	11,4	45,7	31,4
8. Pessoas que morrem por suicídio geralmente têm transtornos mentais.	11,4	34,3	5,7	42,9	5,7
9. É dever de todos tentar impedir alguém de morrer por suicídio.	25,7	57,1	8,6	8,6	-
10. Quando uma pessoa morre por suicídio é algo que ela considerou por muito tempo.	11,4	42,9	22,9	22,9	-
11. Perguntar sobre suicídio pode provocar pensamentos suicidas na pessoa.	-	11,4	17,1	60,0	11,4
12. Pessoas que ameaçam suicídio raramente se suicidam.	-	8,6	20,0	57,1	14,3
13. O suicídio é um assunto sobre o qual não se deve falar.	-	5,7	5,7	37,1	51,4
14. A solidão pode ser para mim um motivo para tirar minha vida.	2,9	14,3	2,9	45,7	34,3
15. Quase todo mundo, em algum momento, já pensou em suicídio	5,7	25,7	14,3	42,9	11,4
16. Pode haver situações em que a única solução razoável seja o suicídio.	-	8,6	5,7	51,4	34,3
17. Eu poderia dizer que me mataria sem ter a intenção de realizar o ato.	5,7	31,4	17,1	25,7	20,0
18. Às vezes o suicídio pode ser um alívio para os envolvidos.	-	28,6	14,3	34,3	22,9
19. Suicídios entre jovens são mais difíceis de entender, pois eles teriam muita vida pela frente.	14,3	45,7	8,6	20,0	11,4
20. Eu consideraria a possibilidade de tirar minha vida se eu sofresse de uma doença grave e incurável.	-	14,3	20,0	34,3	31,4
21. Uma vez que a pessoa teve pensamentos suicidas, nunca deixará de pensar sobre isso.	2,9	14,3	25,7	48,6	8,6
22. O suicídio acontece sem aviso prévio.	11,4	17,1	2,9	60,0	8,6
23. A maioria das pessoas evita falar sobre suicídio.	11,4	74,3	8,6	5,7	-
24. Se uma pessoa quiser se matar, é problema dela e não devemos interferir.	-	5,7	-	37,1	57,1
25. A solidão é o principal motivo que leva as pessoas ao suicídio.	8,6	28,6	17,1	42,9	2,9
26. Uma tentativa de suicídio é essencialmente um grito de socorro.	34,3	51,4	2,9	11,4	-
27. No geral, não entendo como as pessoas podem tirar a própria vida.	25,7	31,4	14,3	25,7	2,9
28. Normalmente, os familiares não têm ideia do que está acontecendo quando uma pessoa está pensando em suicídio.	14,3	54,3	8,6	20,0	2,9
29. Se uma pessoa está sofrendo de uma doença grave, incurável, e expressa o desejo de morrer, deve obter ajuda para isso.	14,3	25,7	17,1	25,7	17,1
30. Me sinto preparado para fazer contato e ajudar uma pessoa que esteja em crise suicida.	5,7	37,1%	14,3	31,4	11,4
31. Qualquer pessoa pode morrer por suicídio.	8,6	40,0	25,7	17,1	8,6
32. É compreensível que pessoas que sofrem de uma doença grave e incurável tirem a própria vida.	2,9	37,1	14,3	34,3	11,4
33. Pessoas que falam sobre suicídio não tiram a própria vida.	-	11,4	25,7	42,9	20,0
34. As pessoas têm o direito de tirar suas próprias vidas.	-	20,0	14,3	31,4	34,3
35. A maioria das tentativas de suicídio é causada por conflitos com uma pessoa próxima.	5,7	20,0	22,9	51,4	-
36. Eu gostaria de obter ajuda para morrer por suicídio se eu tivesse uma doença grave e incurável.	2,9	22,9	17,1	22,9	34,3
37. O suicídio pode ser prevenido.	40,0	51,4	5,7	2,9	-

Os itens que apresentaram maiores escores de discordância com a afirmação foram: item 24 “Se uma pessoa quiser se matar, é problema dela e não devemos interferir” (94,2%); item 13 “O suicídio é um assunto sobre o qual não se deve falar” (88,5%); item 16 “Pode haver situações em que a única solução razoável seja o suicídio” (85,7%); item 6 “Uma vez que uma pessoa tenha decidido morrer por suicídio ninguém consegue impedi-la” (82,9%); e o item 14 “A solidão pode ser para mim um motivo para tirar minha vida” (80,0%) (Tabela 1).

As assertivas que apresentaram maiores escores de indecisão obtiveram um valor de 25,7% de respostas: item 2 “O suicídio nunca pode ser justificado”; item 21 “Uma vez que a pessoa teve pensamentos suicidas, nunca deixará de pensar sobre isso”; e o item 31 “Qualquer pessoa pode morrer por suicídio” (Tabela 1).

## DISCUSSÃO

As etapas deste estudo foram planejadas e conduzidas de acordo com recomendações internacionais reconhecidas e amplamente utilizadas no contexto nacional e internacional para o delineamento de estudos de tradução e adaptação transcultural^(21,24)^. Foram considerados adequados os itens avaliados como adequados por no mínimo 80,0% dos especialistas^(27)^. A definição de valores aceitáveis de IVC confere maior objetividade na análise dos itens^(27)^.

A maioria dos itens obteve IVC superior ao critério estabelecido e os itens que não atingiram esse valor foram aprimorados em uma reunião síncrona com os especialistas, permitindo que a versão final do ATTS-BR fosse criteriosamente construída. O cuidado com as etapas iniciais da tradução e adaptação permitiram a construção de um instrumento avaliado pelo público-alvo como bom, fácil de entender, importante, com opções de resposta compreensíveis e sem necessidade de mudanças ou aprimoramentos.

Alguns participantes afirmaram contato com pessoas que tentaram suicídio (42,9%) ou morreram por suicídio (31,4%). Esses achados podem se relacionar com o tabu associado ao tema, que pode fazer com que as pessoas não reportem pensamentos ou comportamentos suicidas em membros da família a pessoas estranhas, com o intuito de proteger familiares contra possíveis estigmas e evitar a exposição ao tema^(20,29)^.

Os itens com maiores escores de discordância tiveram relação com atitudes desfavoráveis à prevenção, à motivação da pessoa para tentativa de suicídio e ao silenciamento em relação ao tema. As afirmações com maiores escores de concordância tiveram relação com atitudes favoráveis à prevenção e crenças de que o suicídio é um assunto doloroso e pouco discutido. O item 37, por exemplo, avaliou a crença na possibilidade de prevenção e 91,4% dos participantes concordaram que o suicídio pode ser prevenido. Estudos demonstram que pessoas do sexo feminino têm atitudes mais favoráveis à prevenção do que pessoas do sexo masculino^(20)^. Em outro estudo no qual o ATTS também foi utilizado para avaliar as atitudes de profissionais da área de saúde mental que atuam nos cuidados, foi evidenciada elevada concordância com a possibilidade de prevenção do suicídio^(8)^.

Algumas questões do ATTS estão diretamente relacionadas à aceitabilidade do suicídio, uma dimensão atitudinal relacionada a posturas menos condenatórias, mas também à maior propensão ao comportamento suicida e ao menor investimento ou credibilidade nas ações de prevenção^(30,31)^. Estudo realizado na Coreia do Sul demonstrou que atitudes de maior permissividade estiveram associadas à frequência de comportamentos, planos e ideações suicidas, bem como ao risco de tentativas de suicídio^(30)^. Outros preditores associados à aceitabilidade são as questões de gênero, religião e visões políticas^(7)^.

A aceitabilidade do suicídio também é avaliada em situações específicas, como as doenças graves e incuráveis. No presente estudo, a minoria dos participantes concordou que o suicídio é um meio aceitável em situações de doença incurável. Entretanto, na literatura, 61,0% dos participantes concordaram que o suicídio é um meio aceitável no caso de doenças incuráveis, pois a morte nessas condições pode ser avaliada como algo inevitável, enquanto pode ser prevenida em outras situações e/ou circunstâncias^(7)^.

Com relação à associação entre transtornos mentais e suicídio abordada no item 8, 45,7% concordaram e 48,6% discordaram dessa relação. Nem todas as pessoas que morrem por suicídio têm algum transtorno mental e a maioria das pessoas com transtornos mentais não vão a óbito por suicídio^(8)^. Outros estudos reforçam que os profissionais que atuam na área de saúde mental tendem a considerar os transtornos mentais como a principal causa associada ao suicídio^(8)^ e reiteram a concordância sobre a necessidade de aprofundamento sobre a temática^(32)^.

A perspectiva do suicídio como ato de impulsividade abordada no item 4 do ATTS se contrapõe à crença de 48,6% dos participantes. A impulsividade, de forma isolada, não explica completamente o comportamento suicida, mas pode se configurar como fator de risco para tentativas de suicídio, visto que pode ter um papel importante na transição do pensamento para a ação e mediar a progressão da ideação para tentativas de suicídio^(1)^.

O ATTS também avalia crenças sobre a associação entre solidão e suicídio. Observou-se que 37,2% dos participantes concordaram que a solidão é o principal motivo que leva as pessoas ao suicídio (item 25). Diferentes modelos teóricos abordam aspectos interacionais como alguns dos diversos fatores associados ao suicídio, podendo se manifestar como proteção (pertencimento, conexão, relações satisfatórias) ou como fatores de risco (solidão, isolamento, discriminação, violências)^(33,34)^. Embora esses fatores não possam ser entendidos isoladamente no suicídio, a literatura aponta a importância de fortalecer a rede de apoio como estratégia para prevenção do suicídio^(30)^.

No item 23, 85,7% dos participantes concordaram que a “maioria das pessoas evita falar sobre o suicídio” e 42,8% discordaram da afirmativa do item 30 “me sinto preparado para fazer contato e ajudar uma pessoa que esteja em crise suicida”. Essas análises trazem à tona a importância de discussões envolvendo o tema, no intuito de realizar atividades de educação em saúde voltadas para a população. Comumente, o suicídio é um tema evitado e sobre o qual as pessoas se sentem pouco seguras ou confortáveis para dialogar^(35)^. Esse silenciamento ou evitação podem limitar possibilidades de busca de ajuda e implementação de ações preventivas e de cuidado. Falar sobre o assunto de forma assertiva pode ajudar a identificar problemas subjacentes e possibilitar a superação da introspecção durante o diálogo^(8)^.

No contexto atual persistem as crenças de que o suicídio pode ser provocado pelo diálogo sobre o assunto. Neste estudo, a baixa concordância com o item 11 “perguntar sobre suicídio pode provocar pensamentos suicidas na pessoa”, pode sinalizar que há um processo de valorização da importância de falar sobre o suicídio. Nesse cenário, torna-se importante propagar conhecimentos relacionados à comunicação segura sobre o suicídio^(36)^.

Um assunto que precisa ser considerado de forma ponderada e cuidadosa é a possibilidade de perceber sinais indicativos de risco de suicídio. Neste estudo, a porcentagem de concordância com o item 22 (28,5%) se aproximou dos achados de Hjelmeland e Knizek^(37)^, nos quais 29% dos participantes concordaram com a afirmação de que o “suicídio ocorre sem aviso prévio”. Os autores abordam a importância de não subestimar e considerar com seriedade sinais indicativos de risco^(37)^. Contudo, o comportamento suicida não é passível de previsibilidade e alguns sinais de risco podem ser inespecíficos^(38)^.

Estudo realizado a nível nacional evidenciou um aumento nas notificações de violência autoprovocada em todas as regiões do Brasil^(39)^. Essas taxas foram mais frequentes entre mulheres, enquanto os óbitos por suicídio, que também apresentaram aumento em todas as regiões brasileiras, foram mais frequentes em homens na faixa etária de 25 a 59 anos, que contempla grande parte da amostra deste estudo^(39)^. As taxas de suicídio apresentaram um aumento médio de 3.70% ao ano entre 2011 e 2022, sendo que no último ano a taxa foi de 7.3 por 100.000 habitantes^(39)^.

A literatura tem apontado que conteúdos e interações mediadas por tecnologias e a internet parecem exercer influências nas atitudes frente ao suicídio, visto que as pessoas são expostas a uma variedade de informações e ideias e podem interagir de forma diversificada em relação a esse assunto^(7)^. As características de relações entre comunicações virtuais e atitudes relacionadas ao comportamento suicida poderão ser mais exploradas no Brasil após a avaliação das características psicométricas do ATTS-BR.

Espera-se que a adaptação transcultural do questionário ATTS apresente valores psicométricos satisfatórios para sua aplicação, e ofereça mais uma possibilidade de instrumento para avaliação das atitudes frente ao suicídio no contexto brasileiro. Somado a isso, permitirá a análise da influência de fatores sociodemográficos como gênero, cultura e idade nas atitudes frente ao suicídio no contexto nacional.

### Limitações do estudo

Dentre as limitações, destaca-se a amostragem por conveniência no pré-teste, que não permite a generalização dos dados se consideradas especificidades regionais e interseccionalidades. Ressalta-se ainda a necessidade de estudos de validação das propriedades psicométricas do instrumento.

### Contribuições para a área da enfermagem, saúde ou política pública

O instrumento tem potencial para avaliar o efeito de intervenções relacionadas ao letramento em saúde mental, a partir da disponibilização de um instrumento que poderá ser submetido a outras formas de validação e contribuir com discussões, ações educativas, intervenções e políticas que considerem atitudes relacionadas ao suicídio em seu planejamento e escopo de ações.

## CONCLUSÕES

O questionário de Atitudes Frente ao Suicídio foi traduzido e adaptado para o contexto brasileiro por meio de processos recomendados em referenciais internacionais copiosamente utilizados sobre o método. O instrumento passou por etapas de tradução, retrotradução, avaliação de especialistas na temática, bem como avaliação semântica pela população-alvo do estudo. A maioria do público-alvo avaliou a versão final do questionário de forma positiva, considerando os itens importantes, fáceis de entender e sem necessidade de modificações. O instrumento permitiu a análise de fatores que podem influenciar as atitudes frente ao suicídio no contexto nacional, de modo a subsidiar melhores intervenções e ações estratégicas com foco na prevenção do suicídio.

## Data Availability

Os dados da pesquisa estão disponíveis no corpo do artigo.
